# Artificial Polychromatic Light Affects Growth and Physiology in Chicks

**DOI:** 10.1371/journal.pone.0113595

**Published:** 2014-12-03

**Authors:** Jinming Pan, Yefeng Yang, Bo Yang, Yonghua Yu

**Affiliations:** College of Biosystems Engineering and Food Science, Zhejiang University, Hangzhou, 310058, China; Friedrich-Loeffler-Institute, Germany

## Abstract

Despite the overwhelming use of artificial light on captive animals, its effect on those animals has rarely been studied experimentally. Housing animals in controlled light conditions is useful for assessing the effects of light. The chicken is one of the best-studied animals in artificial light experiments, and here, we evaluate the effect of polychromatic light with various green and blue components on the growth and physiology in chicks. The results indicate that green-blue dual light has two side-effects on chick body mass, depending on the various green to blue ratios. Green-blue dual light with depleted and medium blue component decreased body mass, whereas enriched blue component promoted body mass in chicks compared with monochromatic green- or blue spectra-treated chicks. Moreover, progressive changes in the green to blue ratios of green-blue dual light could give rise to consistent progressive changes in body mass, as suggested by polychromatic light with higher blue component resulting in higher body mass. Correlation analysis confirmed that food intake was positively correlated with final body mass in chicks (*R^2^* = 0.7664, *P* = 0.0001), suggesting that increased food intake contributed to the increased body mass in chicks exposed to higher blue component. We also found that chicks exposed to higher blue component exhibited higher blood glucose levels. Furthermore, the glucose level was positively related to the final body mass (*R^2^* = 0.6406, *P* = 0.0001) and food intake (*R^2^* = 0.784, *P* = 0.0001). These results demonstrate that spectral composition plays a crucial role in affecting growth and physiology in chicks. Moreover, consistent changes in spectral components might cause the synchronous response of growth and physiology.

## Introduction

Although the widespread use of artificial light has enhanced the quality of human life and is positively associated with modernity, wealth and security, the rapid global increase in artificial light has fundamentally transformed the light environment over the past six decades in both quantity (6% increase per year, range: 0–20%) and quality (light composition) [1], [2]. Accordingly, the circadian rhythmicity of humans and many domestic animals, when exposed over the long term to variously colored artificial light, is entrained by artificial light instead of typical sunlight.

Therefore, interest has been rising around the mechanisms of individual response to artificial light [3]–[10]. However, though the effects of artificial light on reproductive function [11]–[13], sleep disturbance [14], [15] and mood disorders [16], [17] have started being elucidated, knowledge about the effect of artificial light on the physiological metabolism of individuals is still limited. Moreover, published information on the consequence of spectral composition on individual growth is missing. In addition, previous studies have mainly focused on evaluating artificial light with particular spectral compositions, rather than comparing the effect of artificial light with various spectral compositions on individuals. Currently, the spectral composition of light in urban areas is very diverse, resulting in a mosaic-like spatial distribution of different wavelengths of artificial light systems [13]. As humans and domestic animals are exposed to artificial light throughout their lives, experimental studies on effects of controlled spectral composition on growth and development in humans and domestic animals are urgently needed.

Housing animals in controlled light conditions is useful for assessing the effects of light in animal models. Chickens are one of the best-studied animals with respect to the impact of artificial light. First, the avian retina possesses one of the most sophisticated cone photoreceptor systems among vertebrates. Birds have five types of cones, including four single cones that support tetrachromatic color vision and one double cone, which is thought to mediate achromatic motion perception. Tetrachromatic color vision is mediated by four types of single cones that are maximally responsive to violet, blue, green and red light [18]. Second, they have advanced light receptors within the brain that play an important role in biological and physiological functions [19]–[22]. Third, previous studies have further confirmed that monochromatic green and blue lights can affect growth of chickens [23], [24].

Therefore, in this study, the chicken was selected as the object, and polychromatic light with various green and blue components was selected as the treatments to evaluate the effect of polychromatic light on the growth and metabolism indicators of chickens. In addition, most previous studies focus on a very short time exposure to domestic animals, neglecting the long-term dose of artificial light. As domestic animals are exposed to artificial light throughout their life, chickens were exposed to artificial light treatments from birth to the end of this study.

## Materials and Methods

### Animals and housing

Female chicks (Meihuang#; n = 210, 0 day of age), purchased from a commercial hatchery (Guangda Breeding, Co. Ltd., China), were randomly assigned to five lighting treatments of 30 birds in two replicates of 15 birds. The genetic performance of this medium-growing broiler strain is very stable, and it is certified by the China Agricultural Ministry as one of the two national gene pools of native broiler libraries. All birds were weighed individually at 30 days of age, and the average body mass was calculated immediately for each treatment. To maintain uniformity, 5 birds (such as heaviest, smallest and lame birds) were eliminated per treatment, and 10 broilers from each replicate without creating a deviation from the original average data (20 birds in two replicates of 10 birds from each light treatment group) were reared until the end of the experiment (81 d of age). All chicks had ad libitum access to food and water, and their diets were formulated to meet the nutrient recommendations for poultry (NRC, 1994). All groups were housed in five independent compartments, each of which contained only one light treatment. The independent compartments were divided into 2 sealed cells (replicates) of 1 m^2^ (10 birds alone; 0.1 m^2^/bird). The dry bulb temperature and relative humidity were measured once every day, using data loggers (TH602F, Anymetre Co. Ltd., China) to ensure that the temperature and relative humidity conditions were similar in all compartments. The average environmental temperature and relative humidity were 25.3°C and 67.5%, respectively.

### Experimental protocol

**Figure 1:**
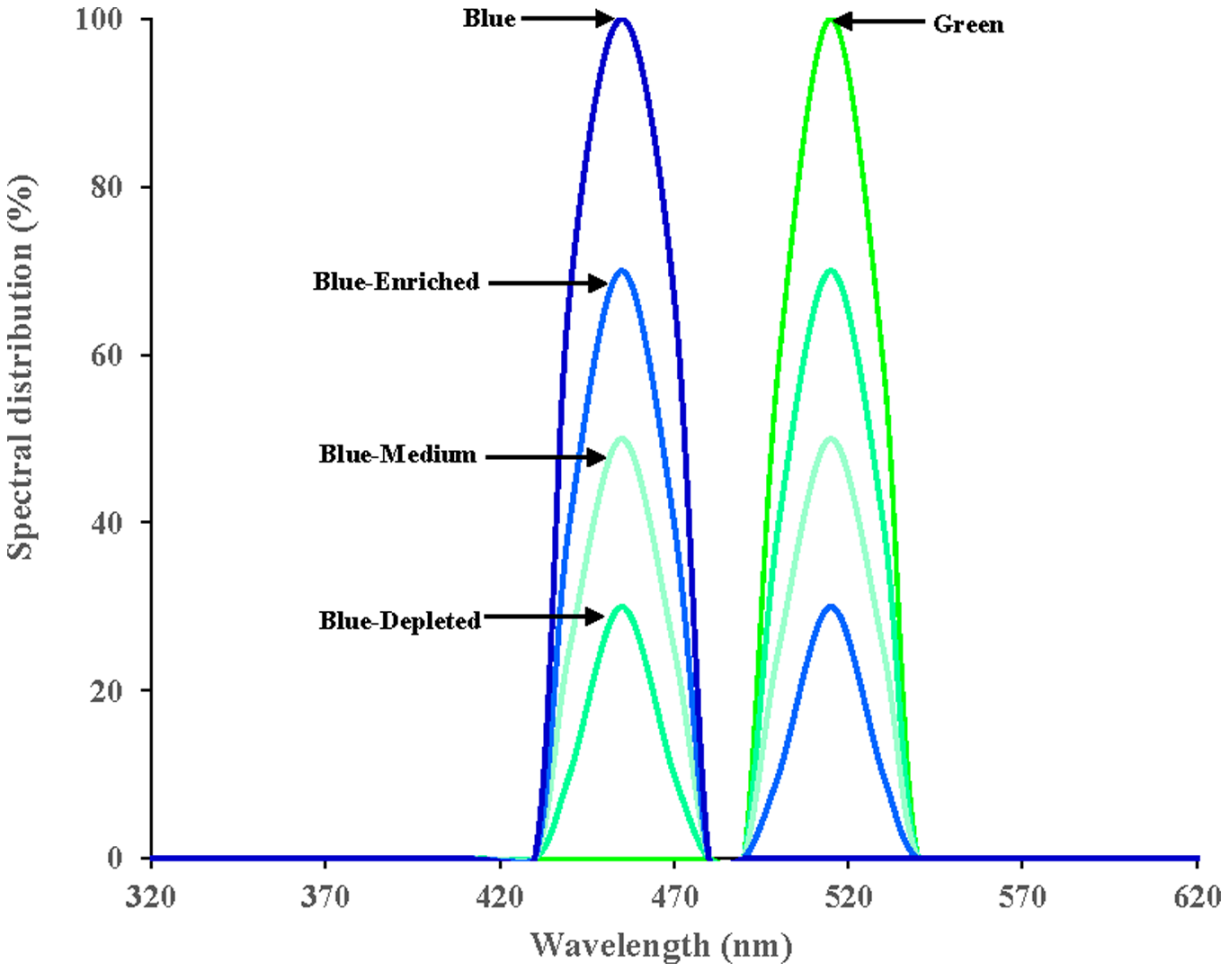
The spectral distribution of the Blue, Blue-Enriched, Blue-Medium, Blue-Depleted and Green LED arrays. The power, current and voltage of each LED array were the same: 2.2 W, 40 mA and 220 V, respectively. Each LED array was controlled by a pulse width modulation (PWM) driver to maintain light intensity at the exact same level.

Upon arrival, all chicks were assigned to one of the following five light spectral groups, including two monochromatic light spectral groups, i.e., the monochromatic green light spectral group (Green group) and monochromatic blue light spectral group (Blue group), and three polychromatic light spectral groups, i.e., the green-blue dual light with depleted blue component (Blue-Depleted group), with medium blue component (Blue-Medium group) and with enriched blue component (Blue-Enriched group). All illumination was provided by light-emitting diode (LED) arrays, purchased from Langtuo Biological Technology Co. Ltd. (Hangzhou, China). The spectral distributions of the LED arrays are presented in Fig. 1. LED arrays of each group were placed 75 cm above the birds using plastic ties attached to the ceiling of compartments. Light intensity was measured as 0.12 Watt/m^2^ of irradiance using a radiometer (AR823, Digital Lux Meter Co. Ltd., China) in each cell at 5 locations at cell floor level. Surroundings were covered with fluorescent fabrics to avoid pollution from other light sources. The light scheduled was 23∶1 light/dark cycle on the first day to make the birds adapt to the environment, and it was reduced by 1 hour every day until it reached 16∶8 light/dark cycle, which was maintained for the remaining days. This study was carried out in strict accordance with the recommendations in the Guide for the Care and Use of Animals of the Zhejiang University. The protocol was approved by the Committee on the Ethics of Animal Experiments of Zhejiang University.

### Measurement of growth and physiology

Food intake and body mass were recorded at 45, 60, 72 and 81 d of age, and the percent gain in body mass was calculated relative to the initial body mass. The growth condition of the seven lighting treatments was described using a nonlinear regression analysis with the mathematical model of body mass vs. age. At the end of the trial (81 days of age), after being fasted for 12 h, 30 birds were randomly selected from each group as 3 birds from each replicate to balance the contribution of the replicates. The birds were then killed by cervical dislocation to collect blood samples and were eviscerated to weigh abdominal adipose. Blood samples were centrifuged at 4°C for 30 min at 3,000×g to remove clots. Blood serum was aspirated and stored in sealable polypropylene micro-centrifuge tubes at −70°C for subsequent determination. Metabolic indicators including total cholesterol (TC), total triglyceride (TG), high-density lipoprotein cholesterol (HDL-CH), low-density lipoprotein cholesterol (LDL-CH) and glucose (GLU) were determined using an Automatic Biochemistry Analyzer (No. AU5400, Olympus Co. Ltd., Japan).

### Statistical analyses

Data were subjected to statistical analyses using SPSS Statistical software (V. 20.). Statistical analysis of data was factorial by rooms and by light. Rooms were found not to be significant for all treated variables, and the results were retested by a one-way ANOVA to analyze the effects of the light spectral composition on birds. Homogeneity of variance was checked for each set of data, and no transformations were applied. When appropriate, post hoc comparisons were made using least significant differences. Data are presented as the mean ± SEM. In every case, a difference between the group means and the correlation coefficient was considered statistically significant if the value of *P*<0.05.

## Results

### Body mass

**Figure 2:**
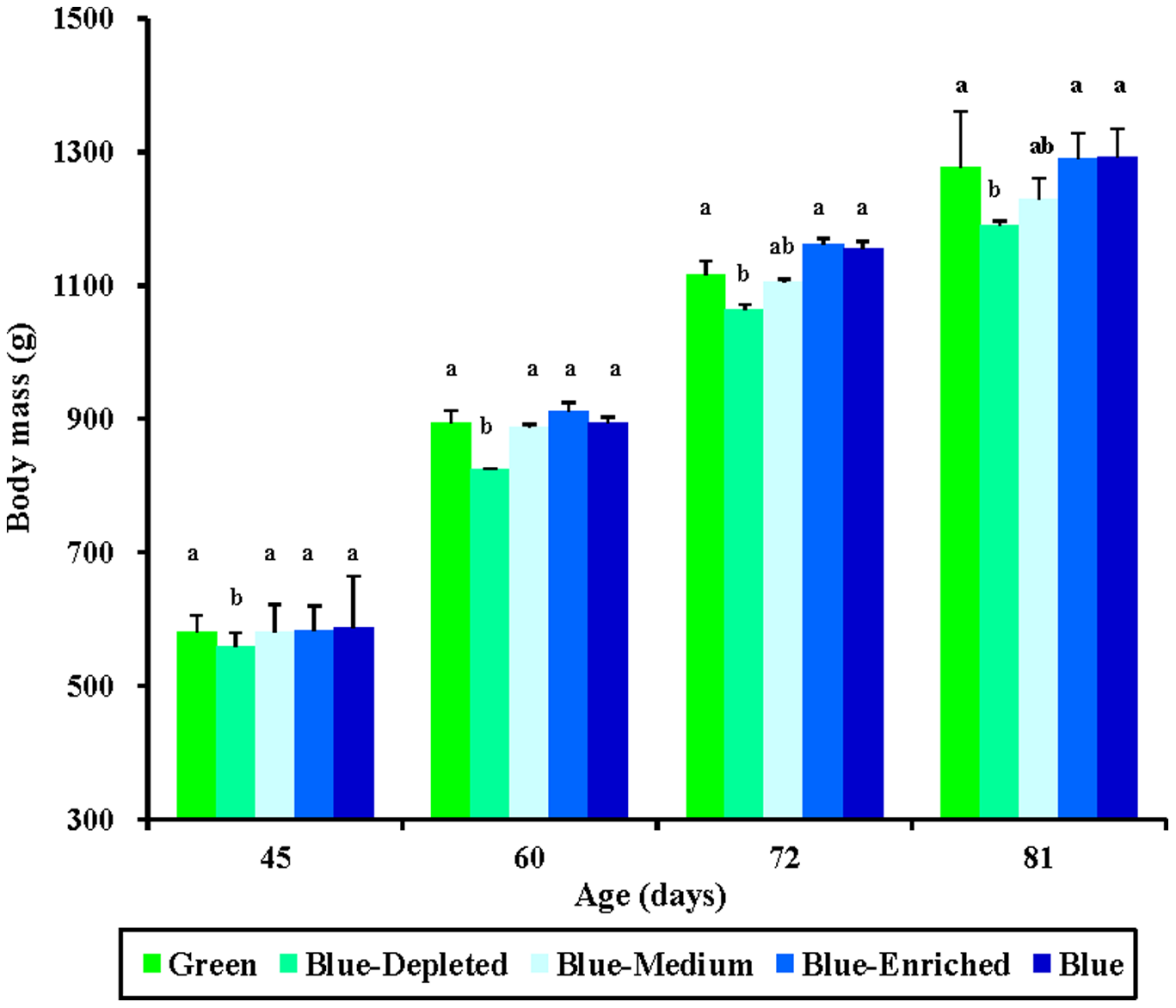
Body mass (g) in chickens reared under different polychromatic light spectra. Each group of treated birds was exposed to either monochromatic light spectra (Green and Blue groups) or green-blue dual light spectra with depleted, medium and enriched blue component (Blue-Depleted, Blue-Medium and Blue-Enriched groups, respectively) from 1 day of age until termination of the experiment at 81 days of age. Body mass was individually measured at 45, 60, 72 and 81 days of age, and the average body mass was calculated. Data are expressed as the mean value ± SEM (n = 20). Bars marked with different letters are significantly different from each other (*P*<0.05).

**Figure 3:**
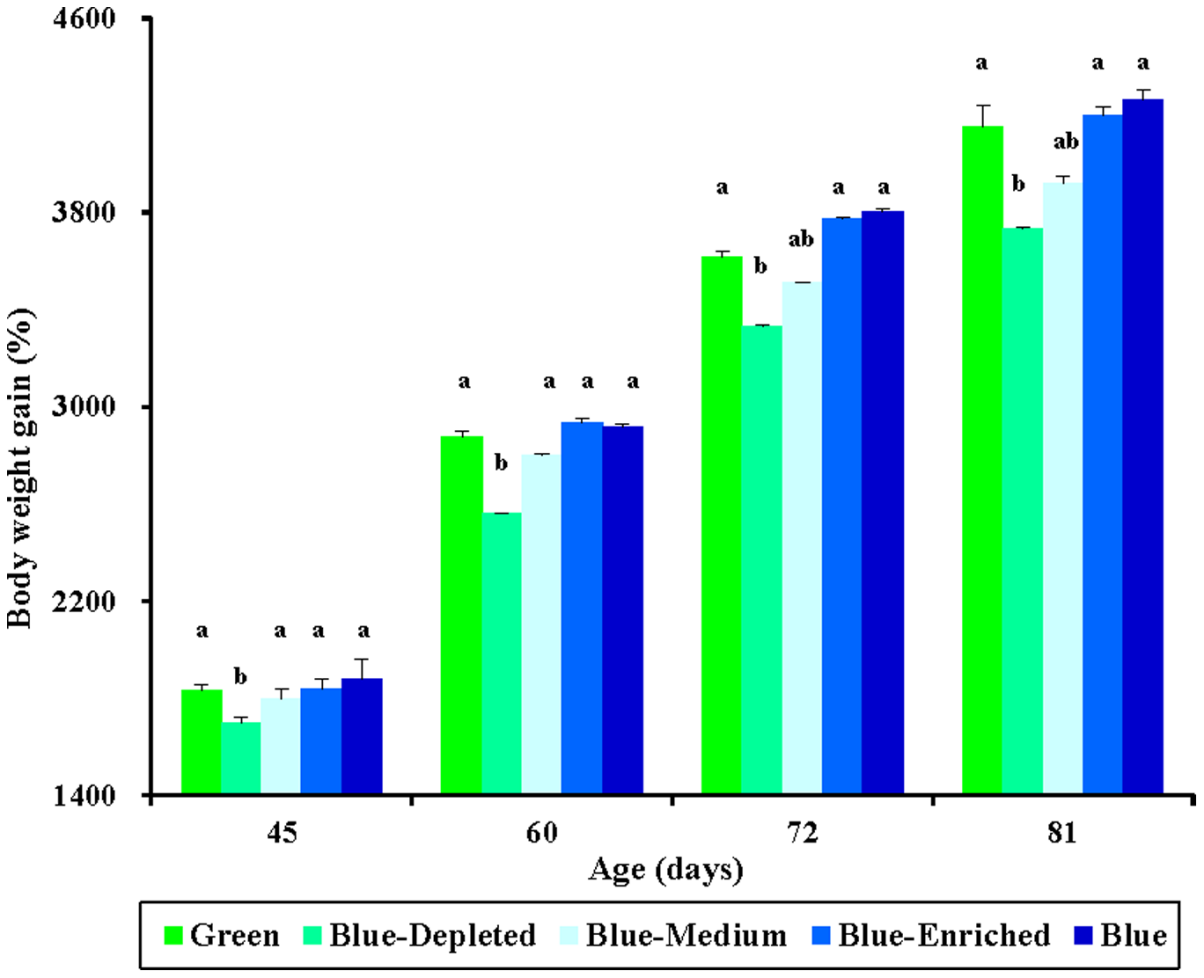
Relative gain in body mass (%, relative to the original body mass) in chickens reared under different polychromatic light spectra. Body mass was measured at 45, 60, 72 and 81 days of age, and the percent gain in body mass (relative to the original body mass) was calculated. Data are expressed as the mean ± SEM (n = 20). Bars marked with different letters are significantly different from each other (*P*<0.05).

The effects of monochromatic light (Green and Blue group) and polychromatic light (Blue-Depleted, Blue-Medium and Blue-Enriched) on the body mass of chicks are given in Fig. 2, which indicates that spectral composition had a significant influence on body mass of birds. A significant decrease in body mass was observed among the Blue-Depleted birds as early as 45 days of age, compared with the Green, Blue, Blue-Medium and Blue-Enriched groups (*P* = 0.028; Fig. 2). This trend continued to 60 days of age. However, at 72 and 81 days of age, no significant difference in body mass was found between Blue-Depleted birds and the Blue-Medium birds (*P* = 0.265). Moreover, at these ages, no significant difference was found among the Green, Blue, Blue-Medium and Blue-Enriched groups (*P* = 0.153). A similar effect of spectral composition was observed for percent body mass gain, as shown in Fig. 3.

**Figure 4:**
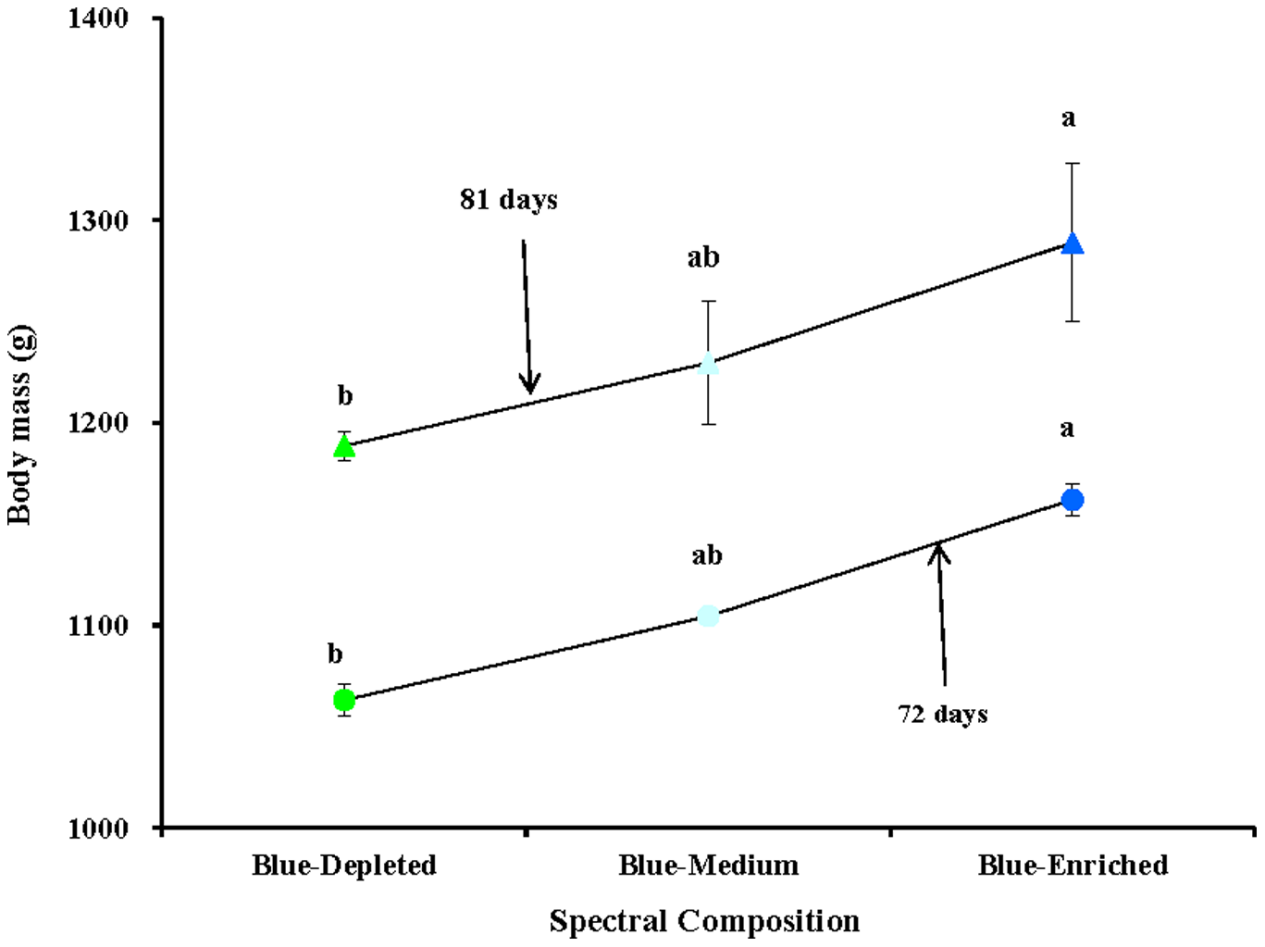
Comparison of body mass (g) in chickens exposed to three blue component levels of polychromatic light spectra: depleted, medium and enriched blue component. Data are expressed as the mean value ± SEM (n = 20). Bars marked with different letters are significantly different from each other (*P*<0.05).

Polychromatic light composition had two-side effects on chick body mass compared with monochromatic light spectra, according to variations in the spectral composition. Thus, relatively lower body masses were obtained in the Blue-Depleted and Blue-Medium groups, while relatively higher body mass values were obtained in the Blue-Enriched group than in the Green and Blue groups. Moreover, we found that polychromatic light with a higher blue component resulted in higher body mass in birds (Blue-Enriched>Blue-Medium>Blue- Depleted groups), as shown in Figs. 2 and 4.

### Food intake

**Figure 5:**
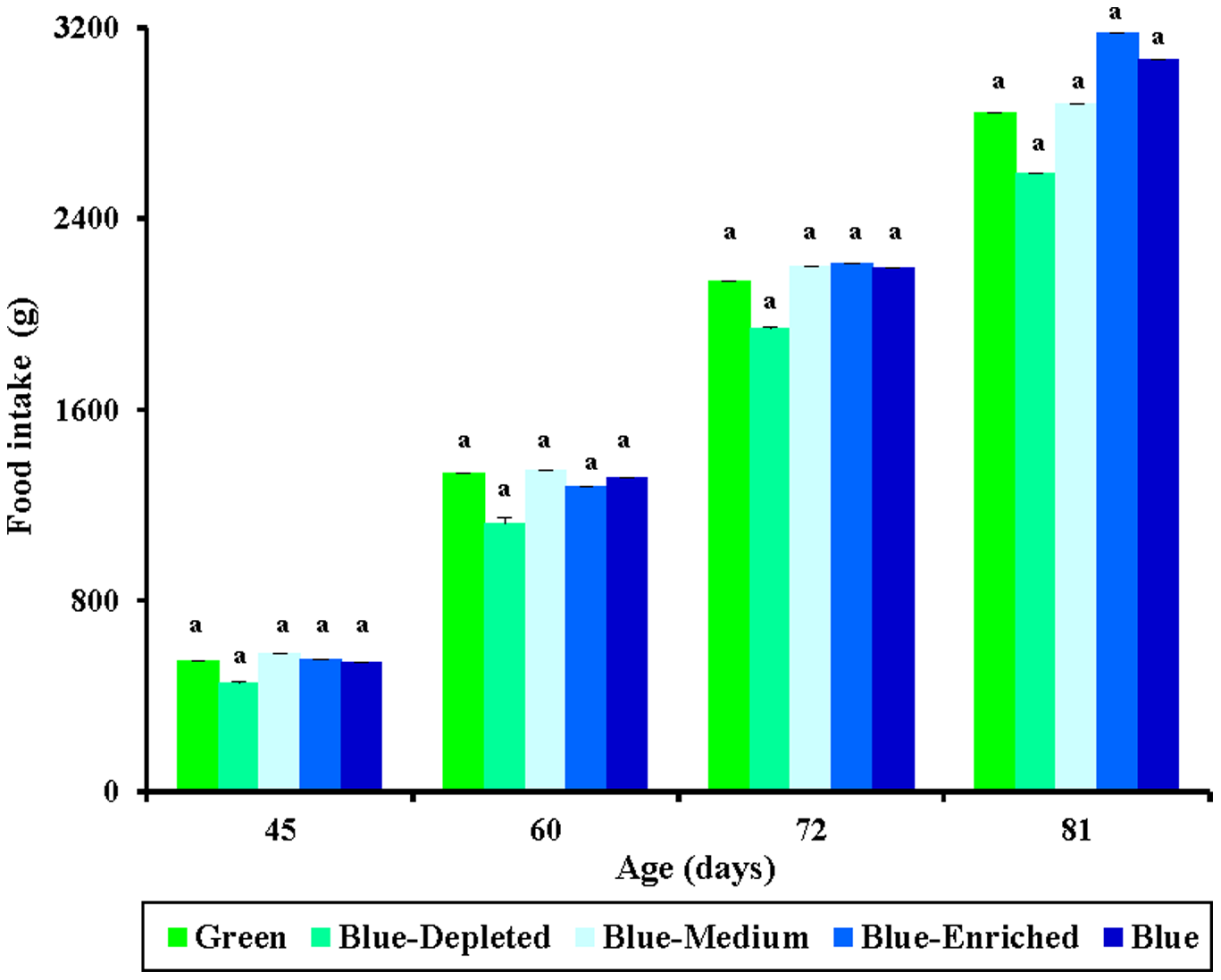
Food intake (g) in chickens reared under different polychromatic light spectra. Food intake was measured at 45, 60, 72 and 81 days of age. Data are expressed as the mean ± SEM (n = 20). Bars marked with different letters are significantly different from each other (*P*<0.05).

**Figure 6:**
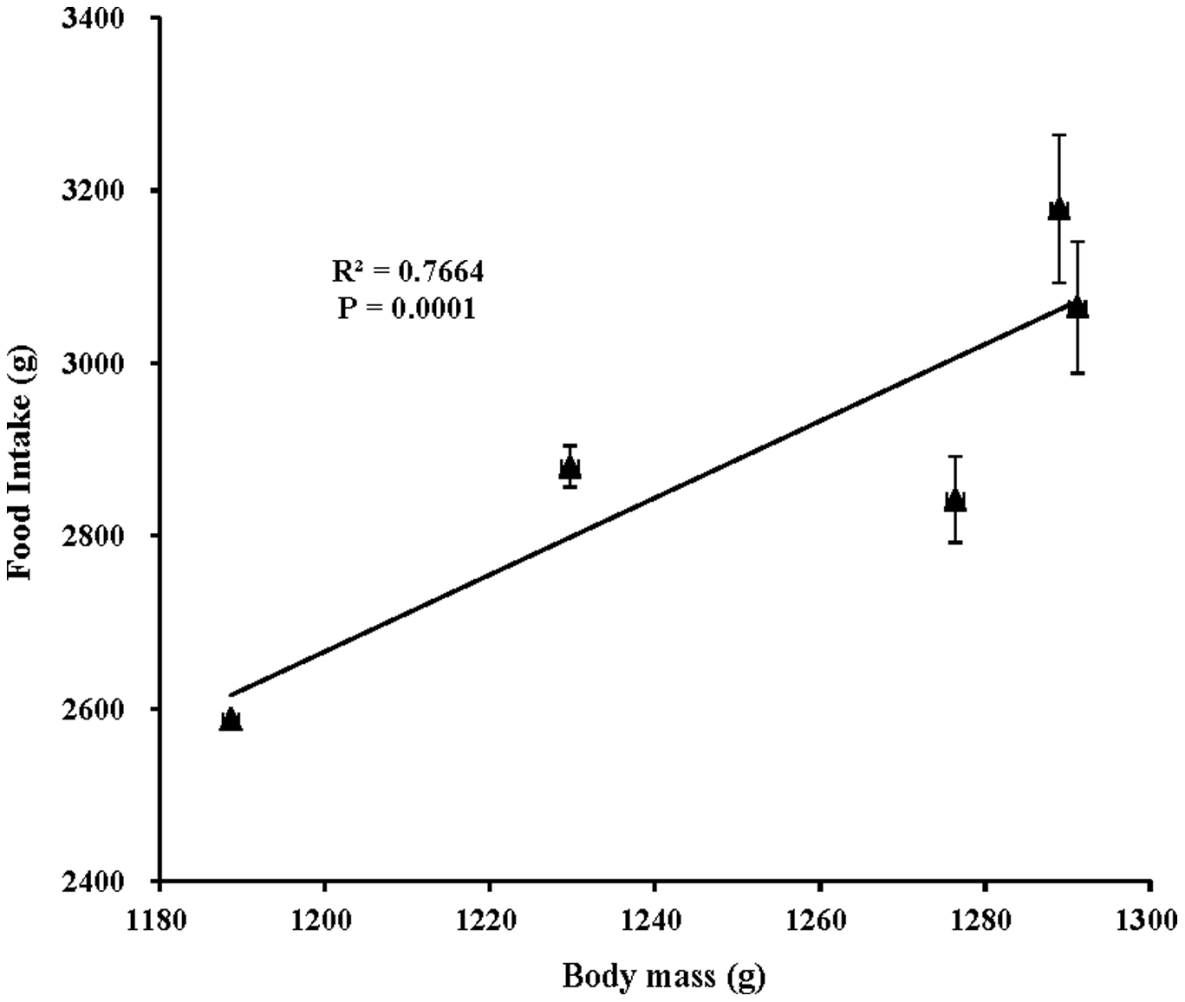
The positive relationship (R^2^ = 0.7664, *P* = 0.0001) between final body mass and food intake of chickens reared under different polychromatic light spectra.

Food intake changed with the growth of the birds such that food intake increased throughout the experimental period (Fig. 5). No significant difference was found in food intake among all lighting-treated chicks (*P* = 0.086). Regardless of age, chicks in the Blue-Depleted group consumed the lowest amount of food compared with the other lighting groups. At 45 days of age, Blue-Medium group chicks ate more food than did the other lighting-treated chicks. This tread continued to 60 days of age. However, at 72 days of age, Blue-Enriched group chicks exhibited the highest food intake of the groups, even higher than that of Blue-Medium group chicks. Moreover, at 81 days of age, chicks exposed to polychromatic light with a higher blue component ate more food than did chicks exposed to a lower blue component, which was consistent with the relationship between the blue component and body mass in chicks (Blue-Depleted<Blue-Medium<Blue-Enriched groups). Correlation analysis confirmed that food intake was positively correlated with final body mass in chicks (*R^2^* = 0.7664; *P* = 0.0001; Fig. 6).

### Food conversion

**Figure 7:**
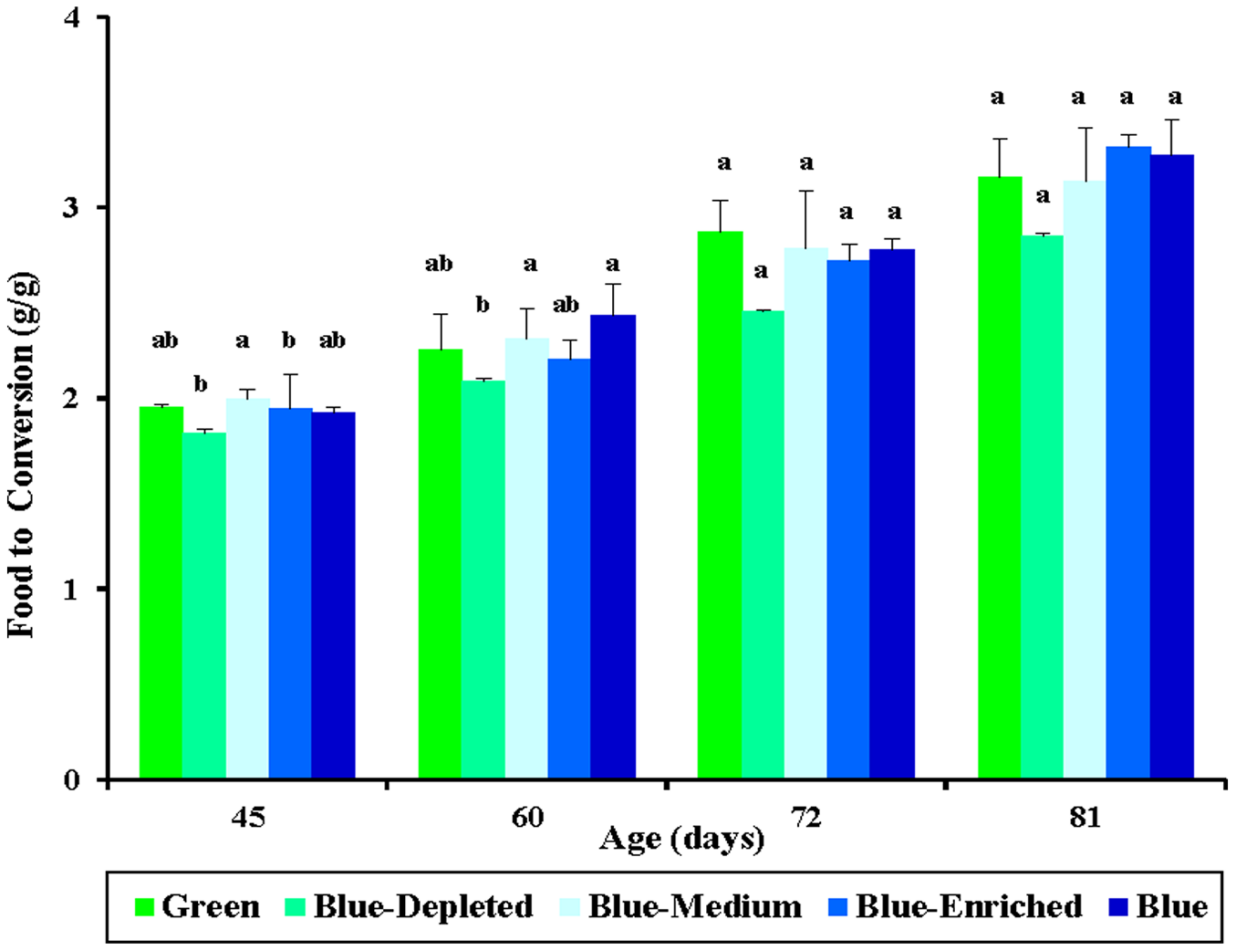
Feed conversion (g/g) of chickens reared under different polychromatic light spectra. Cumulative food consumption and body mass was measured to calculate the food conversion. Feed conversion = (final period body mass – initial period body mass)/period food consumption. Data are expressed as the mean value ± SEM (n = 20). Bars marked with different letters are significantly different from each other (*P*<0.05).

Food conversion can be calculated using the formula (final period body mass – initial period body mass)/period food intake. Food conversion was calculated to directly evaluate the food efficiency of birds in each group. At early growth stages (45 and 60 days of age), a significant lower food conversion in chicks from Blue-Depleted group was found compared with that in Blue-Medium group (*P* = 0.037; Fig. 7). The lowest food conversion value in the Blue-Depleted group and the highest food conversion value in the Blue-Medium group indicated that the highest food efficiency was obtained by Blue-Depleted treated chicks and that the poorest food efficiency was obtained by Blue-Medium treated chicks. However, at a later growth stage (81 days of age), while no significant difference in food conversion was found between chick groups (*P* = 0.081), a positive relationship was observed between blue component and food conversion values (Blue-Depleted<Blue-Medium<Blue-Enriched groups).

### Growth condition

To compare the growth condition of chicks in various lighting groups, their growth curves (body mass vs. age) were adjusted to a nonlinear mathematical model (Gompertz). The Gompertz equation was used in the following form with 3 parameters: W = a×exp(-b×exp(-k×t)), where W is BM (g) at age t (d), a is asymptotic BM reached as t →∞, k is the maturing rate (d^−1^), and ln(b)/k represents the inflection point or maximum growth age [25].

The Gompertz model was quite suitable to describe the growth of birds in all lighting groups (*R^2^* = 0.996, 0.997, 0.997, 0.998 and 0.999 for the Green, Blue-Enriched, Blue-Medium and Blue-Enriched and Blue groups, respectively; Table 1). The spectral composition has little influence on the maturing rate parameter estimates (k) because the parameter estimates k had a narrow range of 0.021–0.026 (d^−1^). However, the estimates of the mature weight (a) and the maximum growth (ln(b)/k) ranged broadly between 1971.3–2658.1 (g) and 55–64 among groups, respectively. Thus, birds from Green group would grow to 2658.1 (g), while birds from Blue group would only grow to 1971.3 (g) as t →∞. Birds exposed to polychromatic light with a higher blue component would result in heavier body mass as t →∞ (Blue-Enriched>Blue-Medium>Blue-Depleted groups). Birds from the Blue-Medium and Blue-Enriched groups would attain maximum growth at 55 days of age, while the Blue-Depleted and Blue groups would attain maximum growth at 56 and 57 days of age, respectively, suggesting that the blue component had a minimal effect on the maximum growth of chicks.

**Table 1:** Parameter estimates obtained by nonlinear regression of body mass vs. age.

group	item^1^	estimate	s.e.m.	P	R^2^
Green	a, g	2658.1	853.8	0.001	0.996
	k,d^−1^	0.021	0.006	0.001	
	ln(b)/k, d	64	0.279	0.001	
Blue-Depleted	a, g	2163.6	431.3	0.001	0.997
	k,d^−1^	0.024	0.005	0.001	
	ln(b)/k, d	56	0.305	0.001	
Blue-Medium	a, g	2197.7	460.0	0.001	0.997
	k,d^−1^	0.024	0.005	0.001	
	ln(b)/k, d	55	0.291	0.001	
Blue-Enriched	a, g	2274.8	325.2	0.001	0.998
	k,d^−1^	0.024	0.004	0.001	
	ln(b)/k, d	55	0.221	0.001	
Blue	a, g	2106.2	297.8	0.001	0.999
	k,d^−1^	0.023	0.003	0.001	
	ln(b)/k, d	57	0.189	0.001	

1 W is body mass (g) at age t (d), a is asymptotic body mass reached as t →∞, and k is maturing rate (d^−1^); ln(b)/k represents the inflection point or maximum growth age, and R^2^ is a measure of regression fit.

### Body temperature

**Figure 8:**
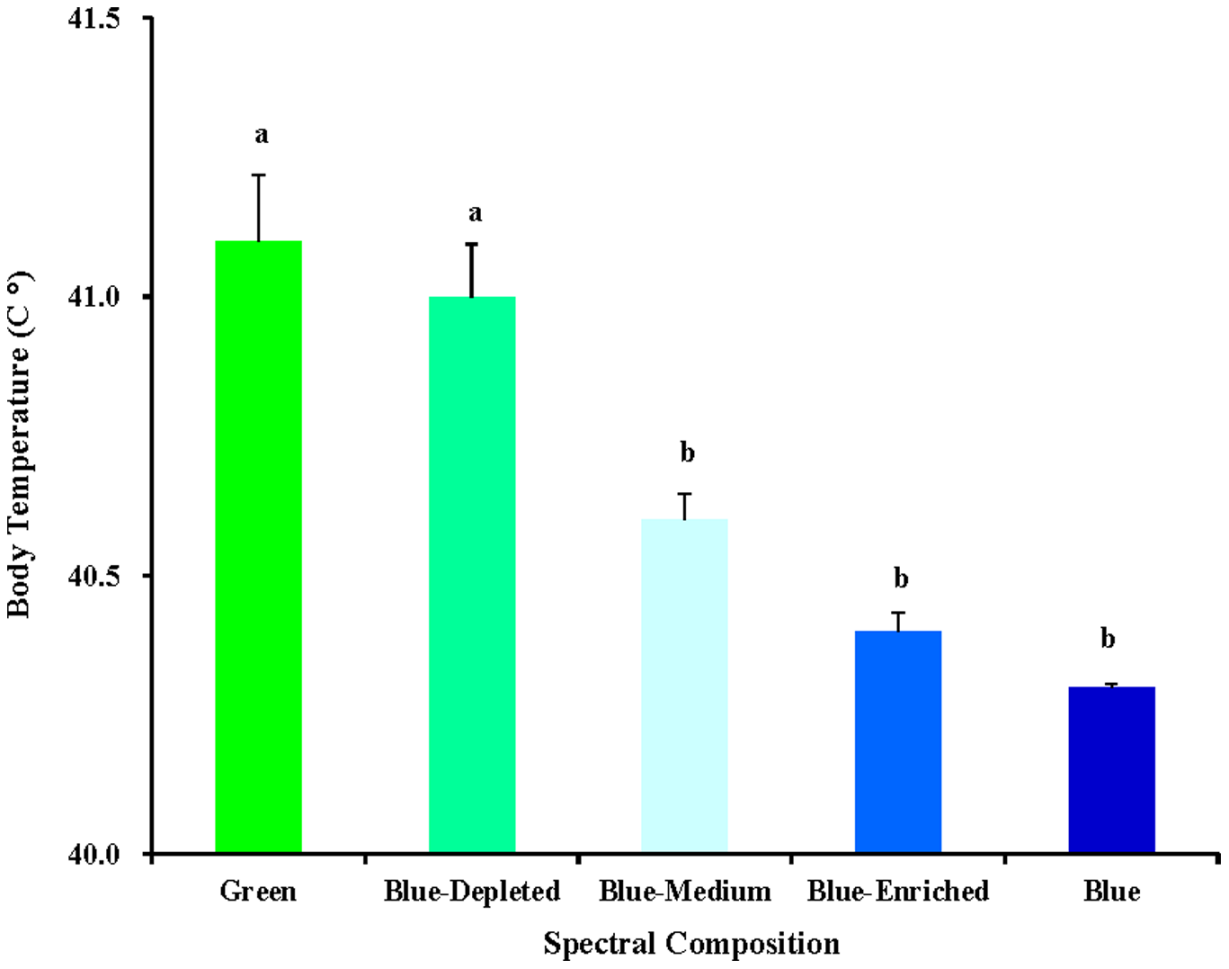
Body temperature (°C) of 81-day-old chickens reared under different polychromatic light spectra. At the end of the trial, body temperature was measured using an infrared thermometer. Data are expressed as the mean value ± SEM (n = 20). Bars marked with different letters are significantly different from each other (*P*<0.05).

The body temperature measurements showed that spectral composition had a significant difference on the body temperature of chicks (Fig. 8). Chicks in the Green group attained significantly higher body temperatures compared with the Blue-Medium, Blue-Enriched and Blue groups (*P* = 0.003). Additionally, chicks in the Blue-Depleted group attained significantly higher body temperatures compared with the Blue-Medium, Blue-Enriched and Blue groups (*P* = 0.015). Moreover, chicks treated with a higher blue component had lower body temperatures (Blue-Depleted>Blue-Medium>Blue-Enriched>Blue groups), suggesting that the body temperature of chicks was negatively correlated with the blue component level.

### Abdominal adipose weight

**Figure 9:**
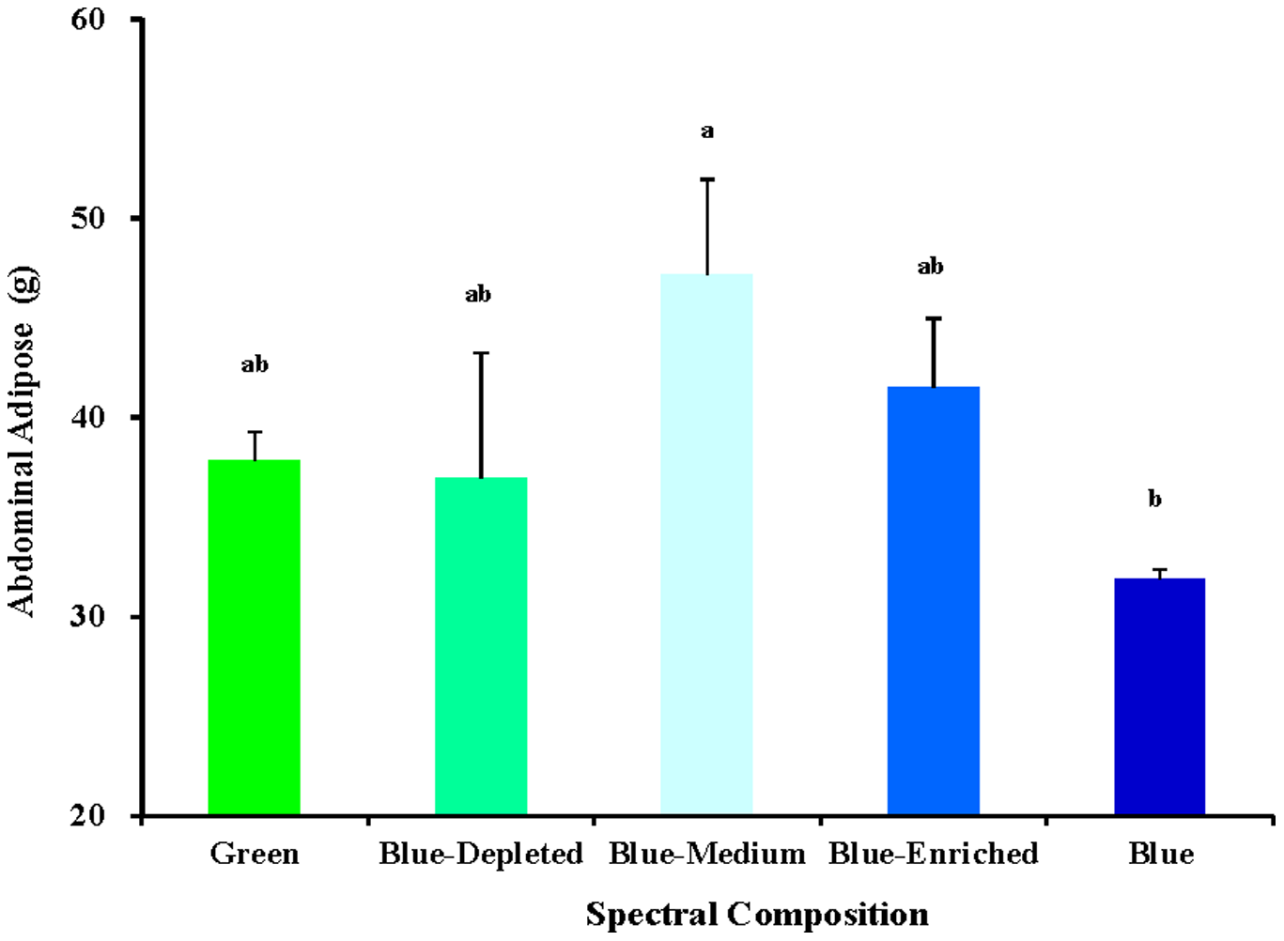
Abdominal adipose weight of 81-day-old chickens reared under different polychromatic light spectra. At the end of the trial, after being fasted for 12 h, birds from each replicate were killed by exsanguination, plucked, and eviscerated to measure weights of abdominal adipose. Data are expressed as the mean value ± SEM (n = 6). Bars marked with different letters are significantly different from each other (*P*<0.05).

As shown in Fig. 9, various spectral-composition treated chicks obtained significantly different abdominal adipose weights (*P* = 0.021). The Blue-Medium group obtained the highest abdominal adipose weight. In contrast to the Blue group, chicks in the Blue-Medium group had significantly higher abdominal adipose weights. However, no significant difference was found in abdominal adipose weight in chicks treated in the Green, Blue-Depleted, Blue-Medium and Blue-Enriched groups (*P* = 0.605).

### Blood metabolic indicators

**Figure 10:**
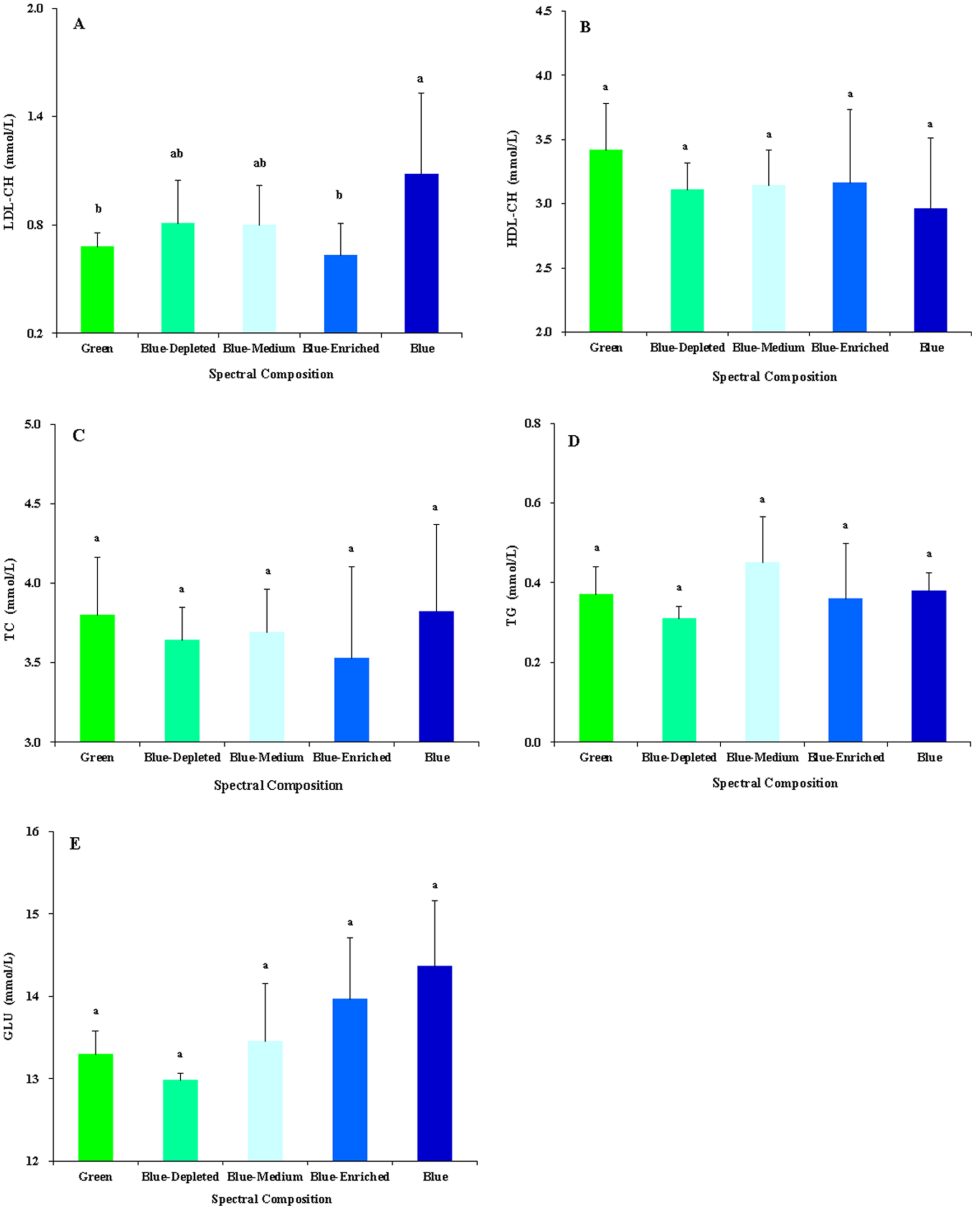
Blood biochemistry parameters (mmol/L) of 81-day-old chickens reared under different polychromatic light spectra. A. low-density lipoprotein cholesterol (LDL-CH), B. high-density lipoprotein cholesterol (HDL-CH), C. total cholesterol (TC), D. total triglyceride (TG) and D. glucose (GLU). Data are expressed as the mean ± SEM (n = 6). Bars marked with different letters are significantly different from each other (*P*<0.05).

**Figure 11:**
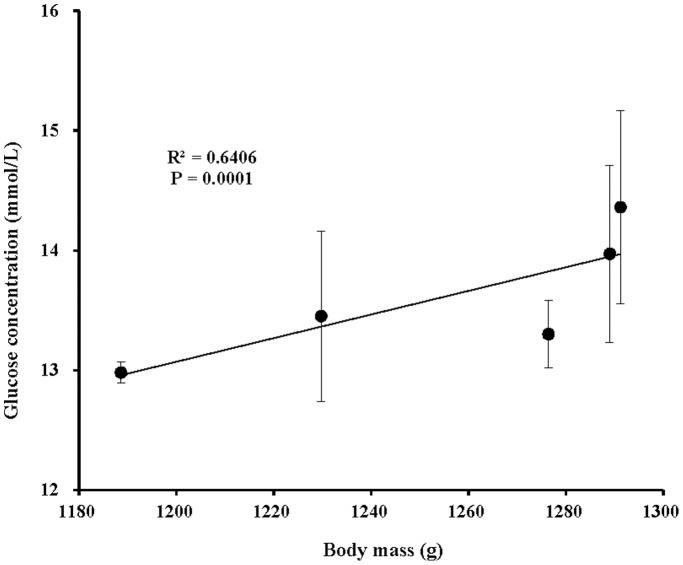
The positive relationship (R^2^ = 0.6406, *P* = 0.0001) between the glucose (GLU) concentration and the final body mass of chickens reared under different polychromatic light spectra.

**Figure 12:**
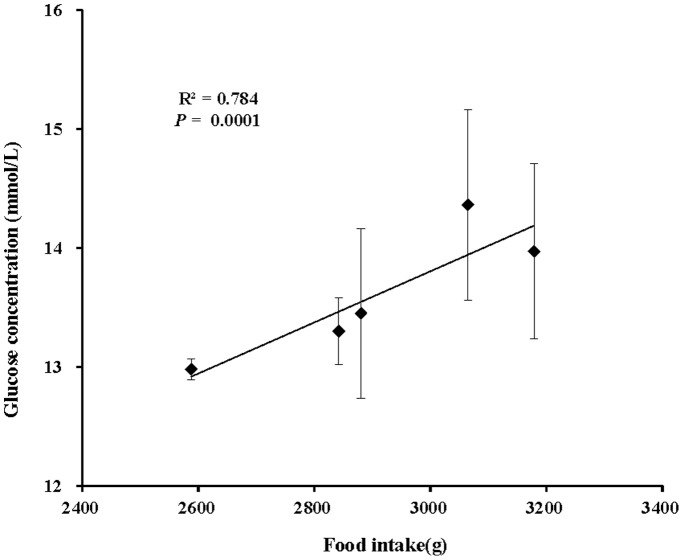
The positive relationship (R^2^ = 0.784, *P* = 0.0001) between the glucose (GLU) concentration and the food intake of chickens reared under different polychromatic light spectra.

The effects of spectral composition on serum metabolic indicators including low density lipoprotein cholesterol (LDL-CH), high density lipoprotein cholesterol (HDL-CH), total cholesterol (TC), total triglyceride (TG) and glucose (GLU) are presented in Fig. 10. Chicks in the Blue group had significantly elevated LDL-CH concentrations compared with the Green group (*P* = 0.047; Fig. 10A). However, though without a significant difference, chicks in the Green group obtained the highest HDL-CH concentrations, whereas this value was lowest in the Blue group (*P* = 0.281; Fig. 10B). Monochromatic spectral group chicks had relatively higher TC concentration than did polychromatic spectral group chicks (*P* = 0.437; Fig. 10C). The Blue-Medium group had a higher TG concentration than did the Blue-Enriched and Blue-Depleted groups (*P* = 0.107; Fig. 10D). As for GLU concentration, chicks treated with a higher blue component had a higher GLU concentration (Blue>Blue-Enriched>Blue-Medium>Blue-Depleted groups) (*P* = 0.057; Fig. 10E), suggesting a positive correlation between the blue component level and the GLU concentration. Furthermore, correlation analyses revealed that the GLU concentration was positively related to the final body mass (*R^2^* = 0.6406, *P* = 0.0001; Fig. 11) and food intake (*R^2^* = 0.784, *P* = 0.0001; Fig. 12).

## Discussion

It has been reported that rates of body weight gain in birds in the tropics are 23% lower than the rates in similarly sized birds inhabiting temperate areas [26]. This difference in weight gain rate could be explained by a difference in lighting duration between the two latitudes, which determines the period during which birds can gather food. However, in the present study, it has been found that light spectral composition, particularly the green and blue component, plays a vital role in affecting growth condition of birds. Moreover, previous reports have shown that blue and green light can promote the growth and development of broiler chickens [27]. It has also been reported that broilers reared under green and blue light combinations had greater body weights than did chickens reared under white light [28]. The present study found that polychromatic light with varying green and blue composition can affect the growth and metabolism of chicks. Specifically, these effects were shown to change according to the blue/green ratio of polychromatic light.

Polychromatic light composition has two-side effects on chick body mass compared with monochromatic light spectra, according to variations in spectral composition. On the one hand, relatively lower body mass values were obtained in the Blue-Depleted and Blue-Medium groups; on the other hand, relatively higher body mass values were obtained in the Blue-enriched group compared with the Green and Blue groups, which indicated that blue-depleted and blue-medium spectra decrease growth and that blue-enriched spectrum promotes growth in chickens. Moreover, progressive changes in the green and blue components could give rise to consistent progressive changes in body mass, as suggested by polychromatic light with higher blue component resulting in a higher body mass in birds (Blue-Enriched>Blue-Medium>Blue-Depleted groups). A previous study reported that artificial light at night (16∶8 light/dim light cycle) can affect body mass [29]. Mice housed in light at night exhibits significantly increased body mass, eating substantially more food at night when exposed to artificial light. Correlation analysis confirms that the increased body mass in mice is contributed to by the food intake at night (*R^2^* = 0.5058, P = 0.006). Therefore, the study suggests that low levels of light at night (5 lux) disrupt the timing of food intake, leading to excess weight gain. The conclusions are relevant to the coincidence between artificial light compositions, especially blue component and body mass increase in birds. As described above, blue component correlated positively with final body mass in chicks. Moreover, chicks exposed to polychromatic light with a higher blue component ate more food than did chicks exposed to a lower blue component. Correlation analysis confirmed that food intake was positively correlated with final body mass in chicks (*R^2^* = 0.7664; *P* = 0.0001). In addition, we also found that light component had a significant influence on abdominal adipose deposition in present study; thus, the Blue-Medium group had the highest abdominal adipose weight among the Blue-treated birds. The chicken is typical of most diurnal birds in that it possesses seven photoreceptor cell types, including one rod and six cones. There are four different types of single cone, which are maximally responsive to violet, blue, green and red light [18]. Prior studies have shown that light energy is converted into neural signals by photochemical changes in the retina [30]. In addition, many other biological responses (e.g. endocrinology [27], immunology [31] and antioxidant capacity [32]) are influenced by light spectra. Therefore, polychromatic light composition can affect the growth of birds directly through the eyes [33].

Food conversion was calculated to directly evaluate the food efficiency of birds in each group. The results of the study indicated that polychromatic light composition with various blue components had a significant influence on feed efficiency, particularly at early ages (45 and 60 days of age). At later growth stages, higher food conversion was found in birds exposed to higher blue component groups, indicating that increasing the blue component could increase the food conversion value (Blue-Depleted<Blue-Medium<Blue-Enriched groups). It was reported that blue light could affect behavior to calm chickens [34] and reduce the activity of the birds [35]. The information given by these reports may explain the higher food consumption and resting condition of the chickens reared under polychromatic light composition with blue component in this study. Therefore, further studies should be conducted to observe the feeding rhythms of chickens and investigate how repose behavior is adjusted to change of light compositions. In addition, fat was needed to produce calories to remain active [36], [37], suggesting that the calming effects of blue light could also contribute to decreased adipose deposition in chicks. In addition, as mentioned above, the lower feed conversion observed with blue light may be related to the biological responses that are induced by blue light, which can enhance the immune response [31] and is known to play a role in promoting intestinal growth [38].

As multiple studies recommended that the Gompertz model be included in growth analyses [25], [39]–[42], the results in present study demonstrated that the Gompertz model was very suitable for describing the birds’ growth in the five light groups. The Gompertz model suggested that the blue component has a minimal influence on the maturing rate parameter, which could affect the mature weight of chicks. Birds from the Green group would grow to 2658.1 (g), while birds from Blue group would only grow to 1971.3 (g) as t →∞, suggesting that asymptotic body mass has the potential to be increased by spectral composition. Moreover, the body mass would be increased as the blue component gradually increased as t →∞ (Blue-Enriched>Blue-Medium>Blue-Depleted groups). Birds from the blue component groups (Blue-Depleted, Blue-Medium, Blue-Enriched and Blue) exhibited the maximum growth at similar growth stages, suggesting that the blue component had a minimal effect on maximum growth of chicks. No comparisons with other reports could be made because no studies have been conducted using growth models to assess the effects of light environment on the growth condition.

The circadian rhythm of body temperature is generated by an endogenous component that is controlled by a circadian clock and an exogenous component that is primarily caused by motor activity variations [43]–[45]. These two functions are related metabolically and temporally [43]. The results in the present study demonstrated that chicks in the Green group obtained significantly higher body temperatures compared with blue component-treated chicks, which demonstrated that body temperature regulation was affected by light components and physiology [46] and that motor activity [47] may be changed by the light component to retain body temperature. Usually, the body temperature of adult fowl is in the range of 40 to 42°C, and significant increases in body temperature occur when environmental temperatures increase significantly [48]. The body temperature measurements in this study ranged from 40.3 to 41.1°C for chicks exposed to variations in the green and blue components, which belonged to the normal body temperature ranges. Moreover, chicks treated with a higher blue component exhibited lower body temperatures (Blue-Depleted>Blue-Medium>Blue-Enriched>Blue groups), suggesting that consistent changes in body temperature corresponded to consistent changes in the blue component level. As mentioned above, blue light has been reported to calm chickens [34] and to reduce the activity of birds [35]. It has also been reported that body temperature is synchronous with locomotor activity in birds [49]. Taken together, the results from these previous reports may explain the reason underlying our current observation of lower body weight and temperature in broiler chickens exposed to light with a greater blue component.

It has been reported that exposing chickens to suboptimal environmental factors, including temperature and light, during the course of growth has an impact on blood physiological variables such as blood acid-base balance and metabolites [50]–[52]. In the present study, we found that artificial spectral composition exerts a significant influence on some blood metabolic indicators but no significant influence on other blood metabolic indicators. Chicks in the Blue group had significantly elevated LDL-CH concentrations compared with the Green group, whereas some indicators including HDL-CH, TG or GLU levels lacked significant differences. Moreover, as reported in the previous study, controlled light environments can reduce much of the hypoglycemia in chickens [53]. In the present study, though without significant differences, the GLU levels in chickens’ blood were gradually elevated as the blue component composition of the exposed light consistently increased. Thus, blue light might be used to alleviate hypoglycemia. In addition, correlation analyses revealed that GLU concentration was positively related to final body mass (*R^2^* = 0.6406, *P* = 0.0001; Fig. 10) and food intake (*R^2^* = 0.784, *P* = 0.0001; Fig. 11).

The mechanisms through which polychromatic light component may affect the growth and metabolism of birds are unclear. However, polychromatic light component may not just have a direct effect through eyes [33], but indirect effects may also exist. Birds are equipped with retinal and extra-retinal photoreceptors [54]. Thus light signals are perceived by the avian brain through eyes (retinas) and direct penetration of skull tissue (extra-retinas) [54]. The electronic method, psychophysical method and behavioral test all indicated that birds (domestic fowl) showed peak sensitivity at blue to green light range (455<λ <571 nm) [55]–[57]. Therefore, polychromatic light with various green-blue components results in different sight sensitivity in chicks. Sight is achieved via the conversion of images that have been formed on the retina into complex electrical signals that are transmitted via the optic nerve to the brain [58]. Although the processes by which the packets (photons) of light energy are converted into neural signals by photochemical changes in the retina are not fully understood, it is probable that many biological responses, such as growth and feed intake, are dependent upon retinal sensitivity. In the present study, data of body mass and food intake of chicks exposed to polychromatic light with various green-blue components differed significantly. Moreover, progressive changes in the green and blue components resulted in consistent changes in sensitivity, giving rise to dose-responsive changes in body mass and food intake. Furthermore, it has been reported that brain photoreceptors communicate directly with gonadotropin-releasing hormone (GnRH) neurons [59] and vasoactive intestinal peptide (VIP) cells [60], [61] that have the potential to determine physiological responses [62], [63] and exert effects through the hormones serotonin and melatonin to affect other endocrine functions [64], including body temperature and blood biochemical parameters. As the results of the present study indicate, body temperature and blood metabolic indicators such as LDL-CH could be significantly affected by light composition. Other indicators such as GLU could even be progressively changed according to consistent change in the exposed light component. Though the exact mechanisms remain unknown, through the two pathways, polychromatic light with various green and blue components entrained the growth indicators, including body mass and food intake, and physiological metabolism indicators, including body temperature and cholesterol. Moreover, progressive changes in the green and blue components could give rise to consistent progressive changes in those parameters.
